# Early Hippocampal Stress Responses to Short-Term Cigarette Smoke Exposure in Mice

**DOI:** 10.3390/ijms27198760

**Published:** 2026-09-30

**Authors:** Rubina Marzagalli, Ng Min Zhen, Christabella Winata, Yik Lung Chan, Venkata Sita Rama Raju Allam, Hui Chen, Brian Gregory George Oliver, Alessandro Castorina

**Affiliations:** 1 Laboratory of Cellular and Molecular Neuroscience, School of Life Sciences, Faculty of Science, University of Technology Sydney, Sydney, NSW 2007, Australia; rubina.marzagalli@uts.edu.au (R.M.); minzhenwendy.ng@uts.edu.au (N.M.Z.); christabella.a.winata@alumni.uts.edu.au (C.W.); 2 School of Life Sciences, Faculty of Science, University of Technology Sydney, Sydney, NSW 2007, Australia; yiklung.chan@alumni.uts.edu.au (Y.L.C.); venkata.allam@slu.se (V.S.R.R.A.); hui.chen-1@uts.edu.au (H.C.); brian.oliver@uts.edu.au (B.G.G.O.); 3 Woolcock Institute of Medical Research, University of Sydney, Sydney, NSW 2113, Australia; 4 Department of Medical Biochemistry and Microbiology, Uppsala University, 752 37 Uppsala, Sweden

**Keywords:** cigarette smoke, hippocampus, endoplasmic reticulum stress, mitochondrial dynamics, oxidative stress, neuroinflammation, neurotoxicity

## Abstract

Cigarette smoke (CS) is a major environmental toxicant associated with cognitive impairment and neurodegenerative disease, yet the earliest hippocampal responses to short-term exposure remain poorly understood. Here, we investigated the effects of short-term CS exposure on hippocampal neuropeptide signalling, endoplasmic reticulum (ER) stress, mitochondrial homeostasis, neuroplasticity and astroglial activation. Mice were exposed to the equivalent of 0, 2, 4 or 6 cigarettes per exposure session, twice daily for four consecutive days, using a whole-body exposure paradigm. Hippocampal tissue was analysed by reverse transcription quantitative PCR, ELISA, immunofluorescence and histology. Short-term CS exposure disrupted hippocampal PACAP/VIP signalling, characterised by reduced *Vip*, *Adcyap1r1* and *Vipr1* expression, decreased VIP protein levels and increased *Vipr2* expression across all smoke-exposed groups, while PACAP remained unaffected. CS also activated ER stress, evidenced by increased phosphorylated IRE1α immunoreactivity and increasing exposure-related trends in *Ern1*, *Ppp1r15a* and *Eif2ak3* expression. Mitochondrial and stress-associated transcriptional responses included increased *Dnm1l*, *Opa1* and *Mmp9* and reduced *Sod1* expression; however, associations among these genes did not remain significant after adjustment for exposure and false-discovery rate. Both *Bdnf* and *Adnp* expression showed a decreasing linear trend without significant individual treatment-versus-control comparisons. Neuronal BDNF immunoreactivity increased in CA1 and CA2/3 without detectable changes in CREB phosphorylation. CS further induced an increasing trend in *Gfap* expression and selective astroglial response within CA2/3. These molecular and cellular responses occurred without overt hippocampal histopathological abnormalities, indicating that brief CS exposure initiates hippocampal stress responses before structural damage is detectable.

## 1. Introduction

Cigarette smoke (CS) is a major source of environmental toxins and a leading cause of preventable morbidity and mortality worldwide [1]. It contains a complex mixture of over 7000 chemicals, including carbon monoxide, tar, nicotine, and a variety of reactive oxidants that have been shown to disrupt cellular and tissue homeostasis across multiple organ systems [2]. While the detrimental effects of CS on pulmonary health are well-established [3], there is increasing recognition of its effects on the central nervous system (CNS), contributing to cognitive impairment [4], neurodegenerative disorders [5,6], and psychiatric illnesses [7]. Importantly, these neurological consequences are not restricted to chronic smokers, as accumulating evidence suggests that even relatively short periods of smoke exposure can initiate molecular disturbances that precede overt neuronal degeneration [8].

Among the brain regions particularly susceptible to CS-induced injury is the hippocampus [9], a structure essential for learning, memory consolidation, spatial navigation, and emotional regulation [10]. The hippocampus comprises anatomically and functionally distinct subfields, including the cornu ammonis (CA1–CA3) regions, dentate gyrus (DG) and subiculum, which coordinate synaptic plasticity and cognitive processing [11]. The high metabolic demand of hippocampal neurons [12], together with their dependence on tightly regulated calcium signalling [13], mitochondrial function [14] and protein quality control [15], renders this region especially vulnerable to oxidative insults and metabolic stress. This region is particularly sensitive to insults such as oxidative stress, altered mitochondrial dynamics, neuroinflammation, and ER stress, all of which are known to be triggered by CS exposure [4,9,16,17].

A cross-sectional MRI study reported smaller total hippocampal and selected subfield volumes in smokers than in non-smokers, together with a stronger association between increasing age and smaller hippocampal volumes among smokers [6]. In a chronic CS-exposure model, mice exhibited impaired working memory together with altered hippocampal microglial and astrocytic profiles, reduced synaptophysin and fewer dendritic spines [4].

The mechanisms responsible for these pathological changes are likely multifactorial. CS generates large quantities of reactive oxygen species that overwhelm the endogenous antioxidant defenses, disrupt mitochondrial bioenergetics and trigger endoplasmic reticulum (ER) stress [18]. These processes are increasingly recognised as interconnected rather than independent events, with ER stress influencing mitochondrial dynamics, inflammatory signalling and cell-fate decisions through the activation of the unfolded protein response (UPR) [19]. Among the three canonical UPR branches, the inositol-requiring enzyme-1α (IRE1α) pathway plays a central role in restoring proteostasis but may also amplify inflammatory signalling when cellular stress becomes excessive [20]. Despite growing evidence implicating these pathways in chronic smoke-induced neurotoxicity, their activation during the earliest stages of exposure remains poorly characterised.

Equally important, neuronal adaptation to environmental stress relies on endogenous neuroprotective systems that have received comparatively little attention in the context of cigarette smoke exposure. The pituitary adenylate cyclase-activating polypeptide (PACAP) and vasoactive intestinal peptide (VIP) signalling network regulates neuronal survival [21,22], mitochondrial integrity [23,24], synaptic plasticity [24] and neuroimmune communication [25] through the PAC1, VPAC1 and VPAC2 receptors. Experimental studies have demonstrated that PACAP/VIP signalling protects the CNS against oxidative stress [26,27,28], excitotoxicity [29] and neuroinflammation [30,31,32], suggesting that disruption of this pathway may represent an early event linking CS exposure to hippocampal dysfunction. Likewise, maintenance of synaptic plasticity depends on neurotrophic signalling mediated by brain-derived neurotrophic factor (BDNF) [33,34] and activity-dependent neuroprotective protein (ADNP) [35], both of which support neuronal resilience, dendritic remodelling and memory formation [36,37,38]. Whether these endogenous protective systems are engaged or become dysregulated during early CS exposure remains unknown.

Despite compelling evidence that chronic smoking damages the hippocampus, little is known about the earliest molecular events that precede structural injury. Most experimental studies have relied on prolonged exposure paradigms lasting weeks to months, making it difficult to distinguish primary stress responses from secondary pathological consequences. Characterising these initial adaptive and maladaptive responses is essential for identifying biomarkers of early neurotoxicity and for defining therapeutic windows before irreversible neuronal damage occurs.

Accordingly, the present study aimed to characterise the early hippocampal response to short-term cigarette smoke (CS) exposure by integrating molecular, biochemical and histological analyses. We hypothesised that repeated short-term CS exposure would induce measurable alterations in hippocampal stress-response and neuroprotective pathways before the emergence of overt histopathological abnormalities. To test this hypothesis, we examined PACAP/VIP neuropeptide signalling, endoplasmic reticulum stress and unfolded protein response markers, mitochondrial-dynamics-associated transcripts, antioxidant and inflammatory responses, neurotrophic signalling and astroglial markers.

Mice were exposed to filtered air or CS equivalent to 2, 4 or 6 cigarettes per exposure session, with two exposure sessions performed daily for four consecutive days, using an inExpose whole-body exposure system. The hippocampus was subsequently assessed using RT-qPCR, ELISA, immunofluorescence and histological analysis to determine whether these molecular and cellular endpoints were altered following short-term CS exposure.

## 2. Results

### 2.1. Short-Term Cigarette Smoke Exposure Differentially Regulates Hippocampal PACAP/VIP Signalling

**Figure 1:**
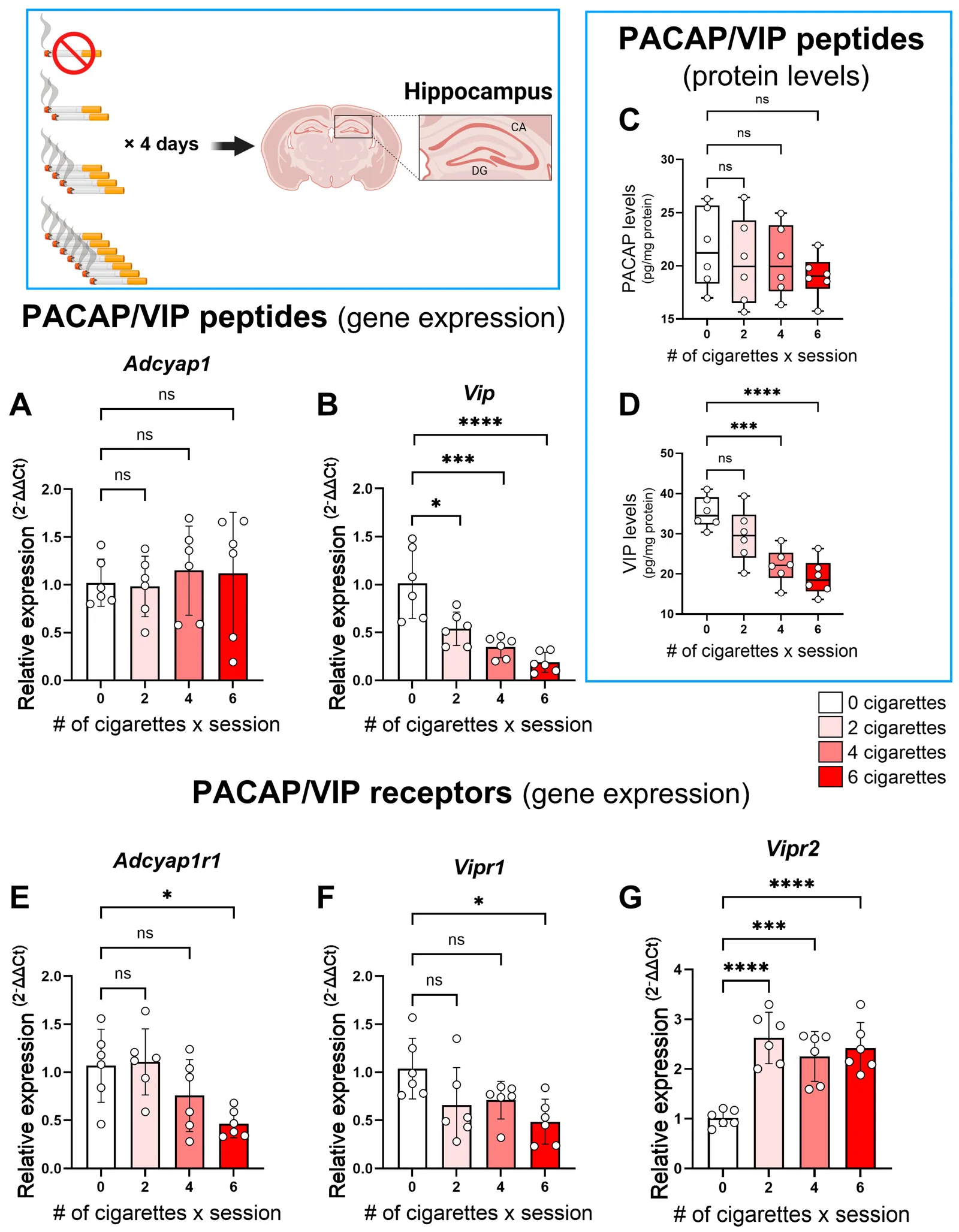
Short-term cigarette smoke exposure differentially regulates hippocampal PACAP/VIP signalling. (**A**,**B**) Relative hippocampal *Adcyap1* and *Vip* expression after exposure to 0, 2, 4 or 6 cigarettes per session, twice daily for four consecutive days. (**C**,**D**) Hippocampal PACAP and VIP protein concentrations quantified by ELISA and normalised to total protein. (**E**–**G**) Relative expression of *Adcyap1r1*, *Vipr1* and *Vipr2*. # in (**A**–**G**) indicates “number”. RT-qPCR fold changes calculated using the 2^−ΔΔCt^ method are displayed, whereas statistical analyses were performed on ΔCt values. Group differences were assessed using one-way ANOVA followed by Dunnett comparisons with control, and linear trends were tested across the ordered exposure levels. Data are mean ± SEM with individual mice shown (n = 6 per group). * *p* < 0.05, *** *p* < 0.001 and **** *p* < 0.0001; ns, not significant.

To determine whether short-term cigarette smoke exposure affects the hippocampal PACAP/VIP neuropeptide system, the mRNA expression of *Adcyap1*, *Vip*, *Adcyap1r1*, *Vipr1*, and *Vipr2*, together with PACAP and VIP protein levels, were quantified in mice exposed to 0, 2, 4 or 6 cigarettes per session, twice daily (Figure 1).

No significant differences were detected in *Adcyap1* mRNA expression among the experimental groups (Figure 1A; one-way ANOVA, *p* = 0.938), and no linear trend was observed across exposure levels (Ptrend = 0.785). In contrast, *Vip* expression decreased significantly with increasing CS exposure (Ptrend < 0.0001; Figure 1B). Compared with control animals, *Vip* expression was reduced in the 2-cigarette group (*p* < 0.05), with larger reductions in the 4-cigarette (*p* < 0.001) and 6-cigarette (*p* < 0.0001) groups.

Consistent with the gene expression data, hippocampal PACAP protein concentrations remained unchanged across all treatment groups (Figure 1C), whereas VIP protein levels were significantly decreased following higher levels of CS exposure (*p* < 0.001 and *p* < 0.0001, respectively; Figure 1D).

The expression of *Adcyap1r1* (PAC1) showed a significant decreasing linear trend across exposure levels (Ptrend = 0.0013; Figure 1E), although the Dunnett comparison with control was significant only in the 6-cigarette group (*p* < 0.05). *Vipr1* (VPAC1) showed a similar decreasing trend (Ptrend = 0.0106; Figure 1F), with a significant reduction confined to the 6-cigarette group (*p* < 0.05).

*Vipr2* expression was significantly increased following CS exposure (one-way ANOVA, *p* < 0.0001; Figure 1G). Relative to control, expression was increased in the 2-, 4- and 6-cigarette groups (Dunnett-adjusted *p* < 0.0001 for the 2- and 6-cigarette groups and *p* < 0.001 for the 4-cigarette group). A significant increasing linear trend was also detected across the ordered exposure levels (Ptrend < 0.001).

### 2.2. Short-Term Cigarette Smoke Exposure Induces Hippocampal Endoplasmic Reticulum Stress and Activation of the Unfolded Protein Response

**Figure 2:**
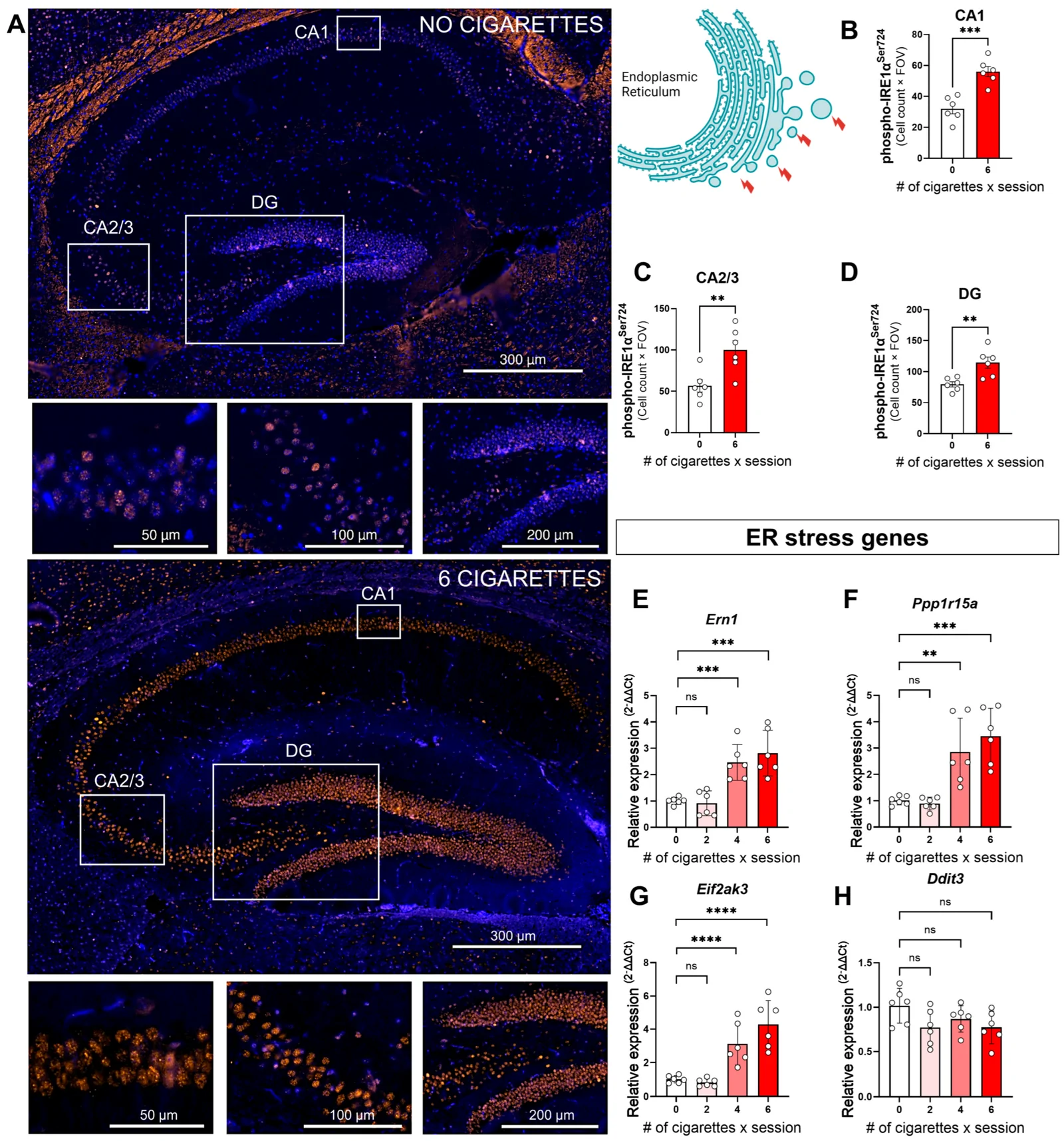
Short-term CS exposure activates ER stress signalling in the hippocampus. (**A**) Representative immunofluorescence images showing phospho-IRE1α Ser724 immunoreactivity in coronal hippocampal sections from control mice and mice exposed to six cigarettes per session twice daily for four consecutive days. Nuclei were counterstained with DAPI (blue). White boxes indicate the CA1, CA2/3 and dentate gyrus (DG) fields shown at higher magnification. Scale bars: whole hippocampus = 300 µm; insets = 50 µm (CA1), 100 µm (CA2/3) and 200 µm (DG). (**B**–**D**) Numbers of phospho-IRE1α-positive cells within predefined CA1, CA2/3 and DG fields of view (FOVs). Each mouse contributed one anatomically matched FOV-derived measurement per hippocampal subregion (n = 6 mice per group). (**E**–**H**) Relative expression of *Ern1* (**E**), *Ppp1r15a* (GADD34) (**F**), *Eif2ak3* (**G**) and *Ddit3* (**H**), displayed as 2^−ΔΔCt^ fold change. Statistical analyses were performed on ΔCt values. Ordinary one-way ANOVA with Dunnett comparisons was used except for *Ern1*, which was analysed using Brown–Forsythe and Welch ANOVA with variance-adjusted comparisons. Linear trends were additionally tested across the ordered exposure levels. Data are mean ± SEM with individual mice shown. ** *p* < 0.01, *** *p* < 0.001 and **** *p* < 0.0001; ns, not significant. IRE1α, inositol-requiring enzyme 1 α; Ern1, Endoplasmic Reticulum to Nucleus Signalling 1; Ppp1r15a, Protein Phosphatase 1 Regulatory Subunit 15A; Eif2ak3, Eukaryotic Translation Initiation Factor 2 α Kinase 3; Ddit3, DNA damage-inducible transcript 3; DAPI, 4′,6-diamidino-2-phenylindole; DG, dentate gyrus; ER, endoplasmic reticulum; UPR, unfolded protein response.

To determine if short-term cigarette smoke exposure triggered ER stress in the hippocampus of mice, we counted hippocampal cells showing immunoreactivity for phosphorylated IRE1α at the Ser724 residue (phospho-IRE1α), a critical activator of the UPR under ER stress [39]. Immunofluorescence analysis demonstrated that CS exposure markedly increased the density of phospho-IRE1α^+^ cells throughout the hippocampus. Representative images showed sparse cells with phospho-IRE1α immunoreactivity in control mice, whereas mice exposed to six cigarettes per session, twice daily, exhibited intense phospho-IRE1α-positive staining across the CA1, CA2/3 and dentate gyrus (DG) regions (Figure 2A). The increase was particularly evident within pyramidal neurons of the CA fields and granule cells of the DG, indicating the widespread activation of hippocampal ER stress signalling.

Quantitative fluorescence analysis confirmed significant regional increases in phospho-IRE1α^+^ cell count, which was significantly increased in the CA1 region (approximately by 2-fold; *p* < 0.001; Figure 2B), CA2/3 and DG (both approximately 2-fold increase; *p* < 0.01; Figure 2C,D).

To further characterise the activation of the unfolded protein response (UPR), the hippocampal expression of *Ern1*, *Ppp1r15a*, *Eif2ak3* and *Ddit3* was quantified across the four exposure groups. *Ern1* expression was unchanged in the 2-cigarette group but increased significantly in the 4- and 6-cigarette groups (both *p* < 0.001; Figure 2E). Because *Ern1* showed unequal variances, the overall group effect was confirmed using Brown–Forsythe and Welch ANOVA (*p* < 0.0001). *Ern1* also showed a significant increasing exposure-related trend (Ptrend < 0.0001). *Ppp1r15a* (GADD34) was similarly unchanged in the 2-cigarette group but increased in the 4-cigarette (*p* < 0.01) and 6-cigarette (*p* < 0.001) groups, with a significant increasing linear trend (Ptrend < 0.0001; Figure 2F).

*Eif2ak3* expression was significantly increased in the 4- and 6-cigarette groups (both *p* < 0.0001) and showed a strong increasing linear trend across exposure levels (Ptrend < 0.0001; Figure 2G). In contrast, *Ddit3* expression did not differ significantly among the exposure groups and showed no significant linear trend (Figure 2H).

### 2.3. Short-Term Cigarette Smoke Exposure Alters Hippocampal Transcripts Associated with Mitochondrial Dynamics, Antioxidative Defence and Extracellular Matrix Remodelling

**Figure 3:**
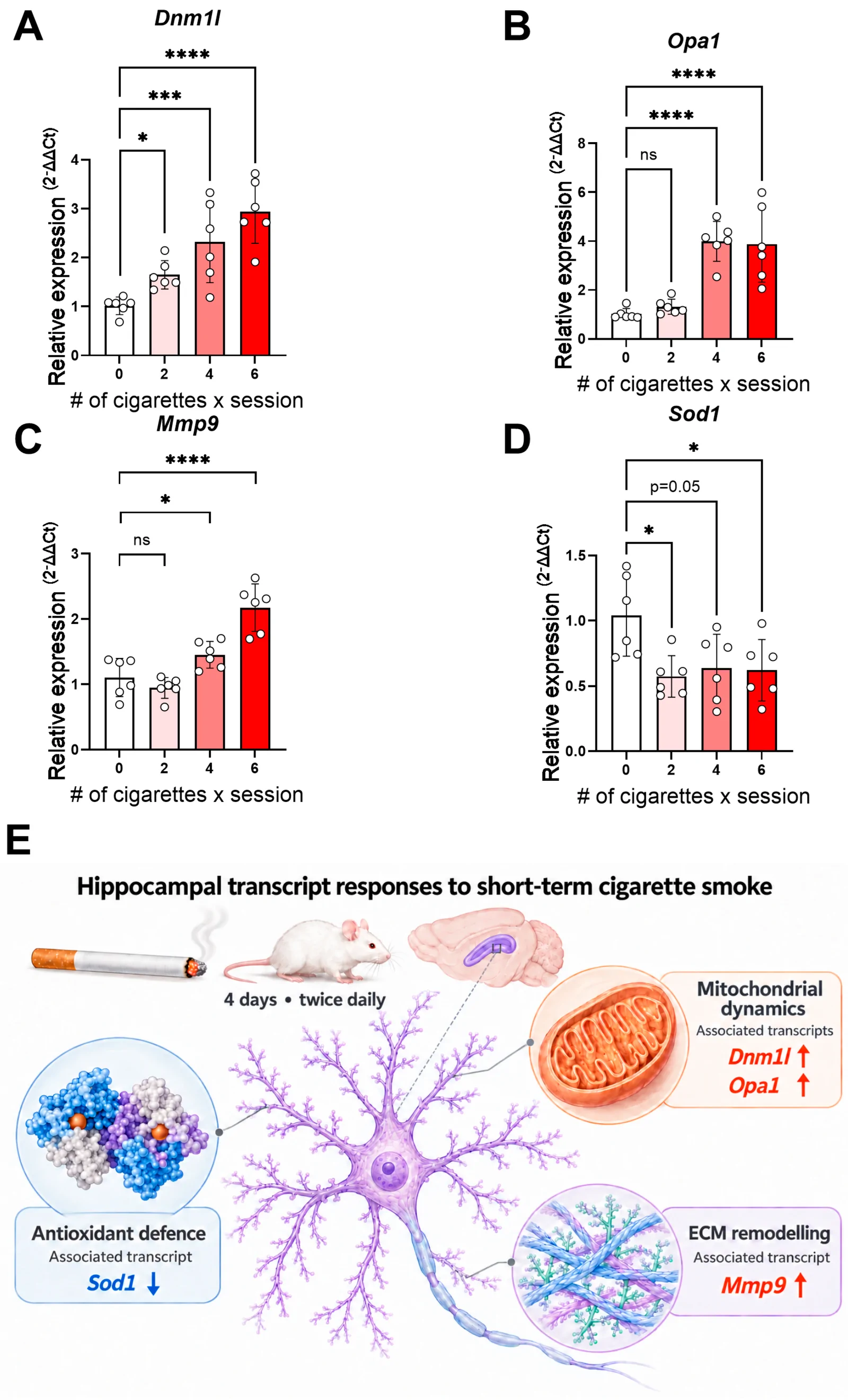
Short-term CS exposure alters hippocampal expression of genes associated with mitochondrial dynamics, extracellular-matrix remodelling and antioxidant defence. Relative hippocampal expression of *Dnm1l* (**A**), *Opa1* (**B**), *Mmp9* (**C**) and *Sod1* (**D**) was measured by RT-qPCR after exposure to 0, 2, 4 or 6 cigarettes per session twice daily for four consecutive days. Fold-change values calculated using the 2^−ΔΔCt^ method are displayed as mean ± SEM with individual mice shown (n = 6 per group), whereas statistical analyses were performed on ΔCt values. (**E**) Schematic summary of hippocampal transcript changes following short-term CS exposure. Increased *Dnm1l* and *Opa1*, increased *Mmp9*, and decreased *Sod1* expression are grouped according to their associations with mitochondrial dynamics, extracellular-matrix (ECM) remodelling and antioxidant defence, respectively. Orange upward and blue downward arrows indicate transcript changes, not measured changes in protein abundance or biological function. The neuronal, organelle and matrix illustrations provide conceptual context; RT-qPCR was performed on hippocampal tissue and does not establish the cellular sources of these changes. Group differences were assessed using one-way ANOVA followed by Dunnett comparisons with control, and linear trends were tested across the ordered exposure levels. * *p* < 0.05, *** *p* < 0.001 and **** *p* < 0.0001; ns, not significant. Exposure-adjusted regression results for the four prespecified transcript associations are reported in Supplementary Table S1.

To characterise exposure-associated transcriptional responses, we quantified hippocampal *Dnm1l* and *Opa1* transcripts, which encode proteins involved in mitochondrial dynamics, *Sod1*, associated with antioxidant defence, and *Mmp9*, associated with extracellular-matrix remodelling (Figure 3).

*Dnm1l* expression increased across exposure levels (Ptrend < 0.0001; Figure 3A). Relative to control, expression was increased in the 2-cigarette group (*p* < 0.05), the 4-cigarette group (*p* < 0.001) and the 6-cigarette group (*p* < 0.0001).

*Opa1* expression also showed a significant increasing linear trend (Ptrend < 0.0001; Figure 3B). Expression did not differ from control in the 2-cigarette group but increased significantly in the 4- and 6-cigarette groups (both *p* < 0.0001).

*Mmp9* expression increased significantly with exposure (Ptrend < 0.0001; Figure 3C), with significant increases in the 4-cigarette (*p* < 0.05) and 6-cigarette (*p* < 0.0001) groups. Conversely, *Sod1* expression showed a decreasing linear trend (Ptrend = 0.0368; Figure 3D). *Sod1* was reduced in the 2- and 6-cigarette groups (both *p* < 0.05), while the 4-cigarette comparison was borderline after Dunnett adjustment (*p* = 0.050).

To determine whether the gene-expression associations persisted beyond their shared relationship with CS exposure, multiple linear regression models included exposure level as a covariate. After exposure adjustment, *Dnm1l* was not independently associated with *Mmp9* (*p* = 0.832), *Opa1* was not independently associated with *Mmp9* (*p* = 0.792), and *Dnm1l* was not independently associated with *Opa1* (*p* = 0.091). The *Sod1*-*Mmp9* association had an unadjusted p value of 0.022 but did not remain significant after Benjamini–Hochberg correction across the four prespecified models (q = 0.090). These exploratory analyses therefore did not support exposure-independent associations among the selected transcripts.

To determine whether short-term CS exposure elicited a classical pro-inflammatory transcriptional response, hippocampal *Il1b*, *Il6* and *Tnf* expression was quantified by RT-qPCR (Supplementary Figure S1). None of the three transcripts differed significantly among exposure groups after Dunnett correction. *Il1b* showed an exploratory linear trend (Ptrend = 0.023), but no individual exposure group differed significantly from control; *Il6* and *Tnf* showed neither significant group differences nor a significant linear trend.

### 2.4. Short-Term Cigarette Smoke Exposure Alters Hippocampal Neurotrophic Signalling

To determine whether short-term CS exposure altered neurotrophic signalling within the hippocampus, neuroplasticity-associated transcripts were quantified by RT-qPCR, followed by an immunofluorescence analysis of BDNF and phosphorylated cAMP response element-binding protein (phospho-CREB Ser133) in coronal hippocampal sections [40].

**Figure 4:**
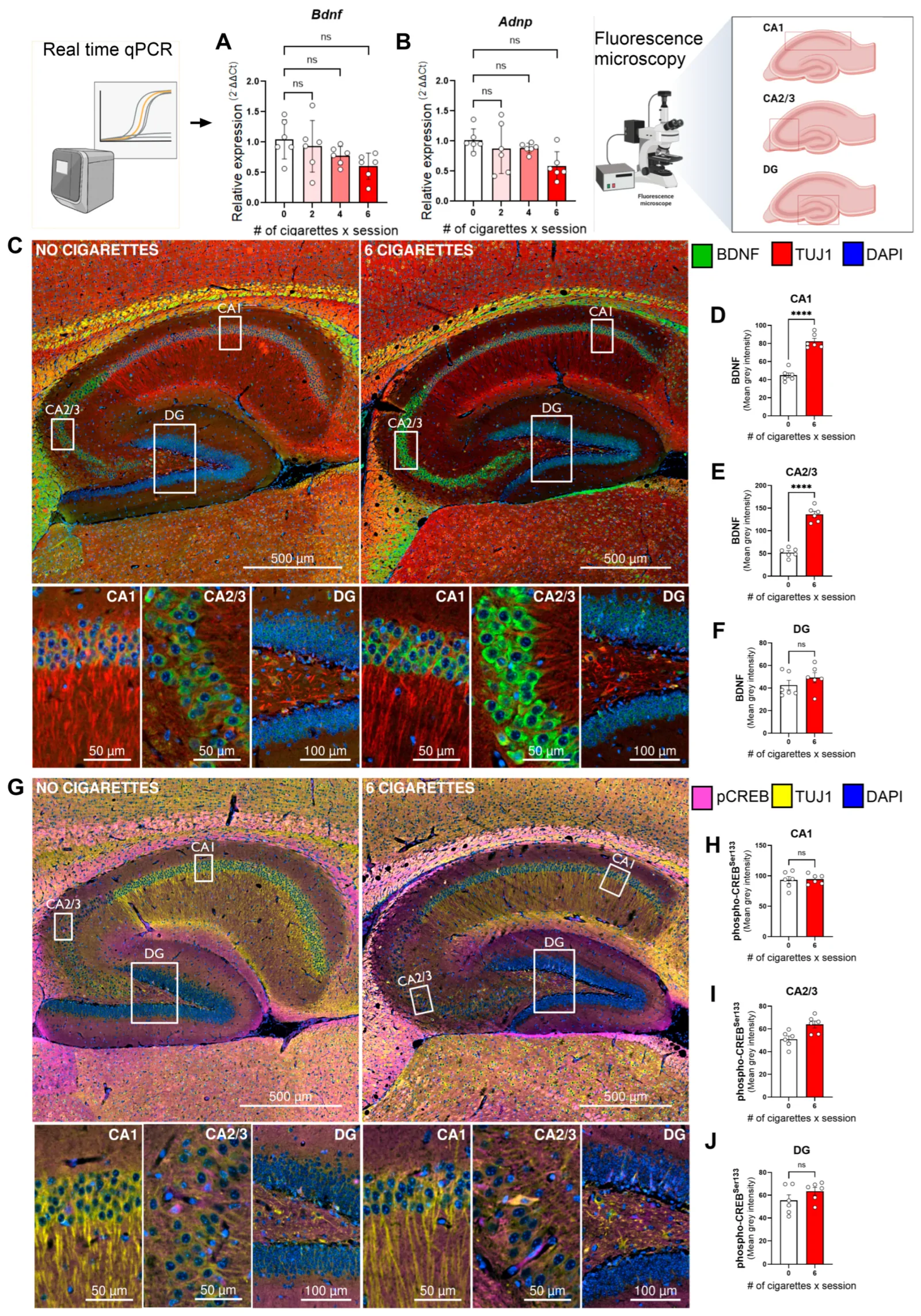
Short-term cigarette smoke exposure differentially regulates hippocampal neurotrophic signalling. (**A**,**B**) Hippocampal *Bdnf* (**A**) and *Adnp* (**B**) expression after exposure to 0, 2, 4 or 6 cigarettes per session twice daily for four consecutive days. Fold-change values are displayed, whereas statistical analyses were performed on ΔCt values. (**C**) Representative immunofluorescence images showing BDNF (green), TUJ1 (red) and DAPI (blue) in coronal hippocampal sections from control and 6-cigarette-exposed mice. White boxes indicate the CA1, CA2/3 and DG fields shown at higher magnification. (**D**–**F**) Background-corrected mean BDNF fluorescence intensity within predefined CA1, CA2/3 and DG FOVs. (**G**) Representative images showing phospho-CREB Ser133, TUJ1 and DAPI in control and 6-cigarette-exposed hippocampi. (**H**–**J**) Background-corrected mean phospho-CREB Ser133 fluorescence intensity within CA1, CA2/3 and DG FOVs. Each mouse contributed one FOV-derived value per region (n = 6 mice per group). *Bdnf* expression was analysed using ordinary one-way ANOVA with Dunnett comparisons, whereas *Adnp* expression was analysed using Brown–Forsythe and Welch ANOVA with variance-adjusted multiple comparisons. Linear trends were additionally tested across the ordered exposure levels. Immunofluorescence comparisons used unpaired two-tailed *t*-tests. Data are mean ± SEM with individual mice shown. **** *p* < 0.0001; ns, not significant. Scale bars: overview = 500 µm; CA1 and CA2/3 = 50 µm; DG = 100 µm. Adnp, activity-dependent neuroprotective protein; Bdnf, brain-derived neurotrophic factor; CA, cornu ammonis; CREB, cAMP response element-binding protein; CS, cigarette smoke; DAPI, 4′,6-diamidino-2-phenylindole; DG, dentate gyrus; IR, immunoreactivity; p-CREB, phosphorylated CREB (Ser133); qPCR, quantitative polymerase chain reaction; RT-qPCR, reverse transcription quantitative polymerase chain reaction; TUJ1, neuron-specific class III β-tubulin (βIII-tubulin).

RT-qPCR analysis showed a decreasing linear trend in *Bdnf* expression across exposure levels (Ptrend = 0.017; Figure 4A); however, the overall one-way ANOVA was not significant (*p* = 0.125), and no individual exposure group differed significantly from control after Dunnett adjustment. Expression levels of *Adnp*—a neuroprotective gene involved in neuronal differentiation and synaptic maintenance [41,42]—also showed a decreasing linear trend (Ptrend = 0.012; Figure 4B); however, no individual exposure group differed significantly from control after variance-adjusted multiple comparisons.

To determine whether these transcriptional changes translated to altered protein localisation within the hippocampus, BDNF immunofluorescence was examined in the CA1, CA2/3 and dentate gyrus (DG) regions. Representative images (Figure 4C) revealed markedly stronger BDNF immunoreactivity in mice exposed to 6 cigarettes per session, twice daily, compared with controls, particularly within neuronal cell layers. High-magnification images further demonstrated that BDNF signal co-localised extensively with the neuronal marker TUJ1, confirming the neuronal localisation of the increased BDNF expression.

Quantitative fluorescence analysis demonstrated that short-term CS induced a region-specific increase in BDNF protein levels. BDNF immunoreactivity was significantly increased in the CA1 region in the 6-cigarette group (*p* < 0.0001 versus control; Figure 4D). An even greater increase was observed within the CA2/3 region (*p* < 0.0001; Figure 4E), where fluorescence intensity more than doubled relative to controls. In contrast, although a modest increase in BDNF staining was evident within the dentate gyrus, this was not statistically significant (*p* > 0.05; Figure 4F).

Immunoreactivity for the downstream target of BDNF, p-CREB^Ser133^, was subsequently examined across the same hippocampal regions (Figure 4G). Representative images demonstrated comparable p-CREB staining patterns between control mice and mice in the 6-cigarette group.

Quantitative analysis confirmed that short-term CS exposure did not significantly modify p-CREB immunoreactivity in any hippocampal subregion. No significant differences were detected in the CA1 region (Figure 4H), CA2/3 (Figure 4I), or DG (Figure 4J), although a modest, non-significant upward trend was observed in CA2/3 and DG. Increased neuronal BDNF immunoreactivity was observed without a statistically significant difference in phospho-CREB^Ser133^ immunoreactivity at the sampled endpoint. This finding does not establish that the BDNF response was independent of CREB signalling.

### 2.5. Short-Term Cigarette Smoke Exposure Induces Mild Astroglial Response in the Hippocampus

**Figure 5:**
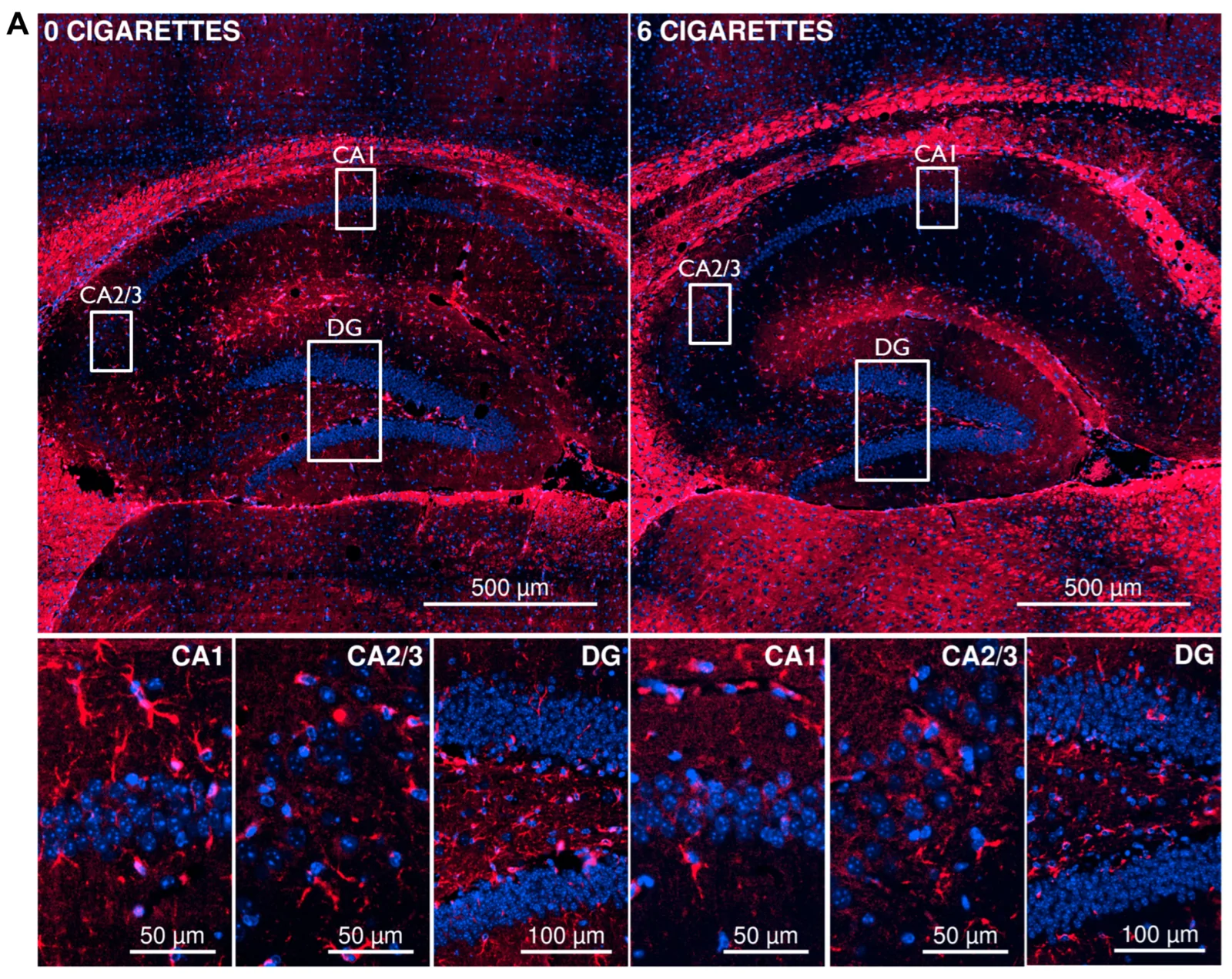

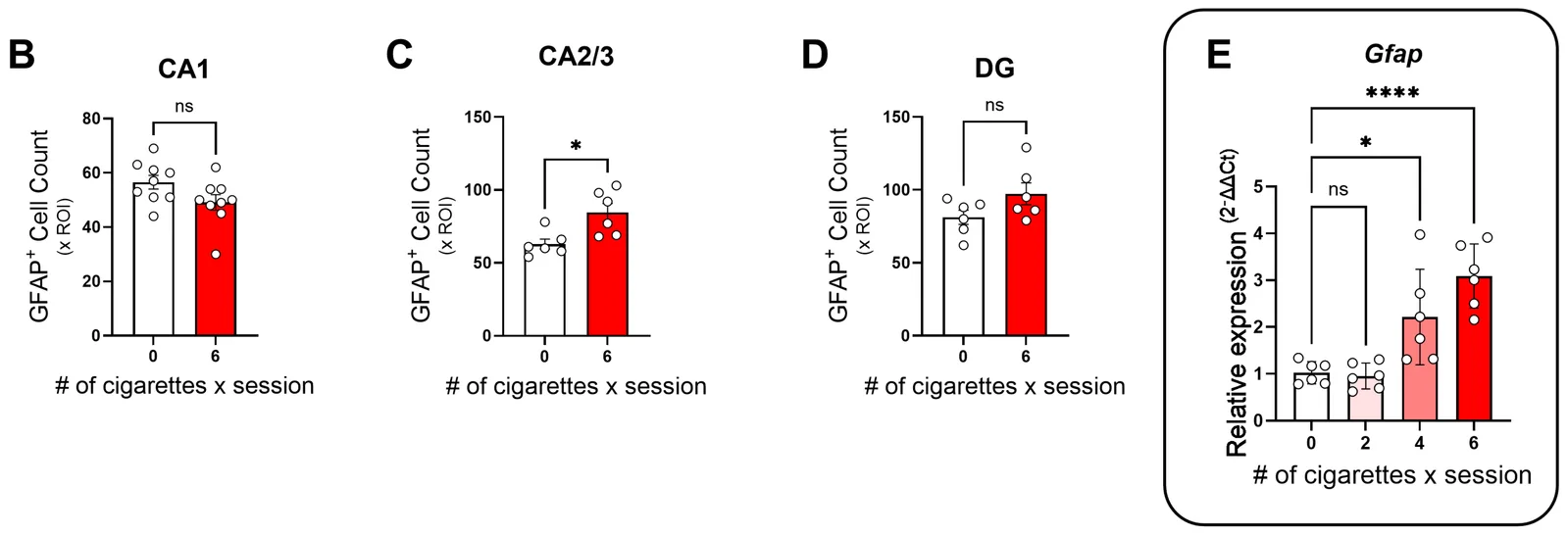
Short-term cigarette smoke exposure increases hippocampal GFAP immunoreactivity and *Gfap* expression. (**A**) Representative GFAP immunofluorescence in coronal hippocampal sections from mice exposed to 0 or 6 cigarettes per session twice daily for four consecutive days. GFAP is shown in red and nuclei were counterstained with DAPI (blue). Higher-magnification images show predefined CA1, CA2/3 and DG FOVs. (**B**–**D**) Numbers of GFAP-positive cells per predefined FOV in CA1, CA2/3 and DG. Each mouse contributed one FOV-derived value per region (n = 6 mice per group). (**E**) Relative hippocampal *Gfap* expression after exposure to 0, 2, 4 or 6 cigarettes per session twice daily for four days. Statistical analyses used unpaired two-tailed *t*-tests for panels (**B**–**D**) and one-way ANOVA with Dunnett comparisons and a linear-trend test for panel (**E**). Data are mean ± SEM with individual mice shown. * *p* < 0.05, and **** *p* < 0.0001; ns, not significant. Scale bars: overview = 500 µm; CA1 and CA2/3 = 50 µm; DG = 100 µm. GFAP, glial fibrillary acidic protein; Gfap, glial fibrillary acidic protein gene; FOV, field of view; CA1, cornu ammonis area 1; CA2/3, cornu ammonis areas 2 and 3; DG, dentate gyrus; RT-qPCR, reverse transcription quantitative polymerase chain reaction; SEM, standard error of the mean.

To determine whether short-term CS exposure induced astrocytic responses, GFAP immunofluorescence was examined in coronal hippocampal sections from mice exposed to 0 or 6 cigarettes per session twice daily for four consecutive days (Figure 5A). Representative images showed GFAP-positive astrocytes across CA1, CA2/3 and DG, with greater immunolabelling most evident in the CA2/3 field of exposed mice.

Counts of GFAP-positive cells within predefined FOVs were significantly increased in CA2/3 (*p* < 0.05; Figure 5C) but did not differ significantly in CA1 or DG (Figure 5B,D). These FOV-derived counts should not be interpreted as stereological estimates of total astrocyte density.

Hippocampal *Gfap* expression showed a significant increasing linear trend across exposure levels (Ptrend < 0.0001; Figure 5E). Expression was unchanged in the 2-cigarette group but increased in the 4-cigarette (*p* < 0.05) and 6-cigarette (*p* < 0.0001) groups compared with control.

### 2.6. Short-Term Cigarette Smoke Exposure Does Not Induce Overt Histopathological Alterations in the Hippocampus

**Figure 6:**
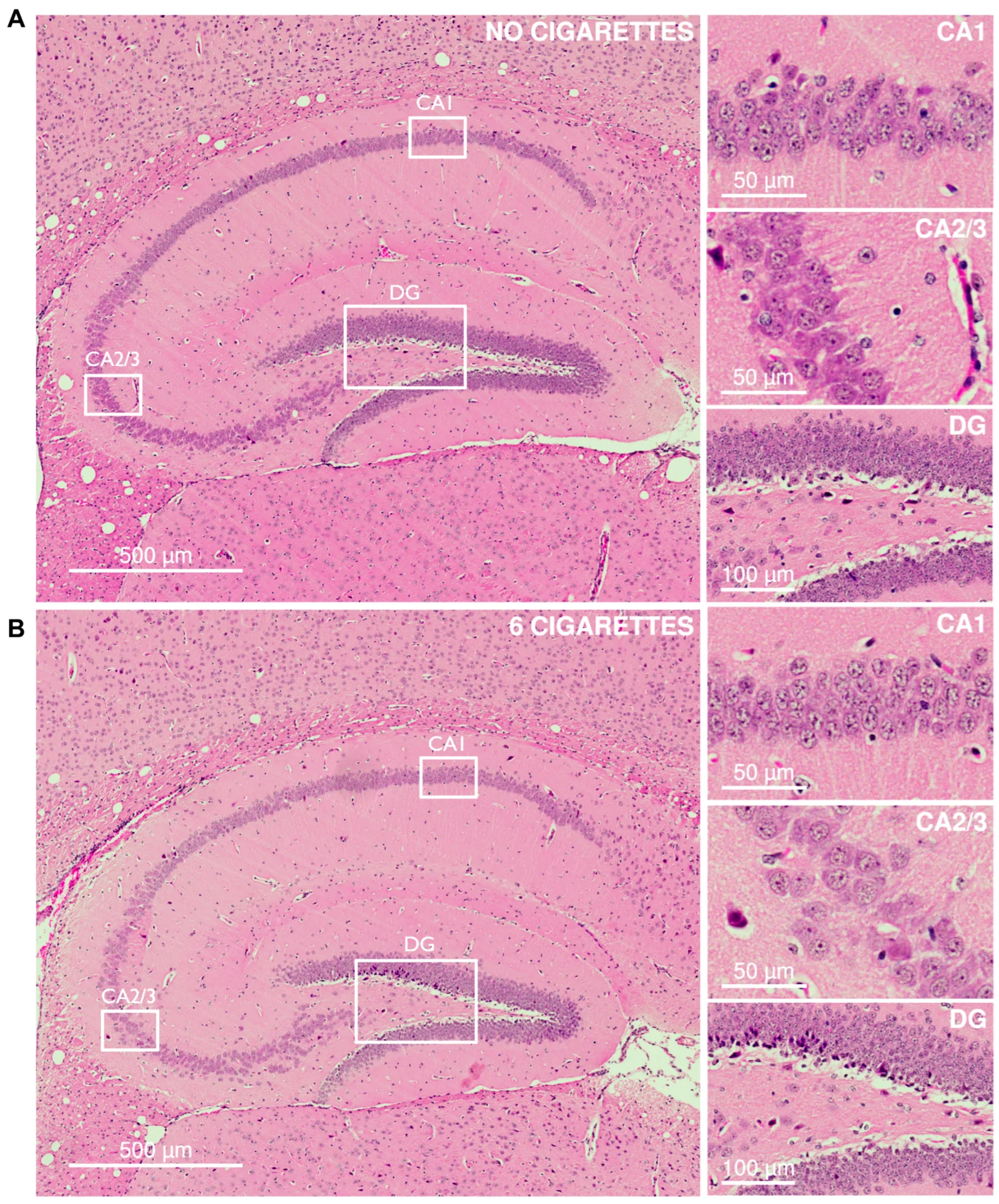
Representative haematoxylin and eosin staining of the hippocampus following short-term CS exposure. Coronal hippocampal sections from mice exposed to 0 cigarettes (**A**) or 6 cigarettes per session twice daily for four consecutive days (**B**) are shown. Low-magnification images demonstrate the sampled hippocampal architecture, with corresponding higher-magnification fields from CA1, CA2/3 and DG. The assessment was qualitative and restricted to overt histological abnormalities. Scale bars: overview = 500 µm; CA1 and CA2/3 = 50 µm; DG = 100 µm.

Representative haematoxylin- and eosin-stained coronal sections from mice exposed to 0 or 6 cigarettes per session twice daily for four consecutive days showed no overt differences in hippocampal architecture at the sampled level (Figure 6A,B).

The CA1, CA2/3 and DG fields retained their expected laminar organisation in both groups, without gross tissue cavitation, haemorrhage or widespread disruption of the pyramidal or granule cell layers.

At higher magnification, no overt neuronal loss or major structural abnormality was apparent in the sampled CA1, CA2/3 or DG fields. Because this assessment was qualitative and did not include formal neuropathological scoring or unbiased stereology, it does not exclude subtle cellular or structural changes.

## 3. Discussion

### 3.1. Short-Term Cigarette Smoke Exposure Disrupts Endogenous Neuroprotective Signalling and Activates Early Endoplasmic Reticulum Stress Responses

Short-term CS exposure is known to induce oxidative stress and inflammation [18,43], yet the earliest molecular events preceding overt neuropathology remain poorly understood. The present study demonstrates that only four days of CS exposure are sufficient to trigger coordinated changes in hippocampal neuroprotective signalling and ER stress pathways, despite the absence of detectable histopathological abnormalities. These findings support the concept that molecular dysfunction develops before structural injury and suggest that the hippocampus responds rapidly to environmental toxicants by engaging multiple stress-response pathways. Notably, these early molecular alterations occurred in the absence of detectable changes in the expression of the classical pro-inflammatory cytokines *Il1b*, *Il6* and *Tnf*, indicating that the disruption of endogenous neuroprotective pathways and activation of ER stress precede the onset of a robust inflammatory response. This temporal sequence suggests that oxidative and proteostatic stress represent some of the earliest hippocampal adaptations to short-term CS exposure, with neuroinflammatory signalling likely emerging only during more prolonged or severe exposure.

Among the most notable findings was the selective disruption of the PACAP/VIP signalling system [44,45,46]. *Vip*, *Adcyap1r1* and *Vipr1* showed decreasing exposure-related trends, whereas *Vipr2* was increased across all three smoke-exposed groups. PACAP expression and protein abundance remained unchanged. The sustained *Vipr2* increase may represent a compensatory regulatory response to reduced VIP availability, although its functional significance remains to be established [47,48]. Together, these findings identify VIP-associated signalling as a sensitive early target of CS exposure.

CS exposure also produced a clear activation of ER stress signalling, as demonstrated by increased IRE1α phosphorylation and an elevated expression of *Ern1*, *Eif2ak3* and *Ppp1r15a* (GADD34). These observations are consistent with previous studies showing that CS activates the unfolded protein response (UPR) in peripheral tissues and extend these findings to the hippocampus after short-term exposure [49]. Importantly, *Ddit3* (CHOP), a key mediator of ER stress-induced apoptosis, remained unchanged. This pattern suggests the activation of an early UPR aimed at restoring proteostasis rather than initiating cell death [50,51]. Similarly, increased *Ppp1r15a* expression is compatible with the engagement of the negative feedback mechanisms that promote the recovery of protein synthesis following transient ER stress.

The disruption of neuroprotective peptide signalling, UPR activation and altered mitochondrial gene expression occurred concurrently after short-term CS exposure. These parallel responses are compatible with a broader cellular stress response [52,53], but the present expression data do not establish direct mechanistic coupling among the pathways.

### 3.2. Mitochondrial-Dynamics-Associated Transcriptional Changes, Altered Neurotrophic Markers and Astroglial Responses

The hippocampus is particularly vulnerable to oxidative stress because of its high metabolic demand and dependence on mitochondrial function [14,24]. Short-term CS exposure increased *Dnm1l* and *Opa1* expression and reduced *Sod1* expression, supporting an early transcriptional response involving mitochondrial dynamics and antioxidant defence [54,55]. Because no direct measurements of mitochondrial morphology, fission, fusion or redox status were performed, these findings should be interpreted as pathway-associated transcriptional changes rather than the direct evidence of functional mitochondrial remodelling or oxidative injury.

Increased *Mmp9* expression identifies an exposure-associated transcriptional change in a gene encoding an extracellular-matrix-remodelling enzyme; it does not demonstrate increased matrix remodelling or MMP9 activity. MMP9 participates in extracellular matrix remodelling and synaptic plasticity under physiological conditions and has also been implicated in neuroinflammation and blood–brain barrier dysfunction following sustained injury [56,57]. Because MMP9 protein abundance, enzymatic activity and cellular source were not assessed, the transcriptional increase should not be interpreted as the direct evidence of classical neuroinflammation or blood–brain barrier disruption.

A particularly interesting observation was the differential pattern of *Bdnf* transcript and neuronal BDNF protein expression. Although *Bdnf* expression showed a modest downward trend with increasing CS exposure, the overall group effect was not significant, and no individual exposure group differed significantly from the control group. In contrast, BDNF immunoreactivity increased within hippocampal neurons, particularly in the CA1 and CA2/3 regions. This apparent difference between the transcriptional and protein-level responses may reflect post-transcriptional regulation or the transient intracellular retention of BDNF during acute cellular stress [58,59]. Although the underlying mechanism remains uncertain, these findings indicate that early CS exposure may influence neurotrophic signalling primarily at the protein level during this short exposure period. Phospho-CREB Ser133 immunoreactivity did not differ significantly between groups at the sampled endpoint. However, the single-time-point design cannot exclude earlier, transient or otherwise undetected CREB responses, and the present data do not establish whether CREB contributed to the increased BDNF immunoreactivity.

Astrocytes also responded rapidly to CS exposure, as shown by increased *Gfap* expression and enhanced GFAP immunoreactivity. Reactive astrocytes play an important role in maintaining neuronal homeostasis during oxidative stress by regulating extracellular glutamate, ion balance and metabolic support [60,61]. Interestingly, astroglial response was most evident within the CA2/3 region, suggesting that hippocampal subfields differ in their sensitivity to acute smoke exposure. While the mechanisms responsible for this regional specificity remain unclear, this observation is consistent with the recognised functional and metabolic heterogeneity in hippocampal circuits.

Taken together, these findings show that short-term CS exposure is associated with changes in transcripts related to mitochondrial dynamics and antioxidant defence, alongside changes in neurotrophic and astroglial markers. The exposure-adjusted regression analyses did not demonstrate independent associations among *Dnm1l*, *Opa1*, *Mmp9* and *Sod1* after false-discovery-rate correction; therefore, the data support concurrent exposure-related responses rather than a demonstrated coordinated mechanistic axis.

### 3.3. Early Hippocampal Molecular Responses: Implications, Limitations and Future Directions

Despite the molecular changes observed in the hippocampus, qualitative histological assessment showed no overt architectural abnormality after four days of CS exposure. This finding indicates that gross structural damage was not detectable under the sampling and staining conditions used, but it does not exclude subtle cellular or ultrastructural alterations.

The coexistence of changes in neuropeptide-signalling markers, ER-stress-associated endpoints, mitochondrial-dynamics-associated transcripts and astroglial markers suggests an early hippocampal response to CS exposure despite preserved tissue architecture [51,62]. Although the present study does not establish whether these changes are ultimately protective or contribute to later pathology, they identify several pathways that may represent early indicators of hippocampal vulnerability. Defining how these molecular responses evolve during prolonged CS exposure will be important for determining when reversible cellular dysfunction progresses to irreversible neuronal injury.

Several limitations should be considered when interpreting these findings. First, behavioural testing was not performed, preventing a direct assessment of whether the observed molecular alterations are accompanied by deficits in hippocampal-dependent learning and memory. Second, the study focused on selected molecular pathways rather than unbiased transcriptomic or proteomic approaches, which may reveal additional mechanisms involved in the early response to CS exposure. Third, only male mice were examined. As sex influences oxidative stress responses, neuroinflammation and mitochondrial function, future studies should determine whether similar molecular adaptations occur in females. Finally, the present study examined a single early time point. Longitudinal studies incorporating behavioural assessment, electrophysiological measurements and advanced molecular profiling will be important for defining the temporal relationship between early molecular changes, synaptic dysfunction and structural neurodegeneration.

Overall, this study demonstrates that short-term CS exposure is sufficient to alter multiple hippocampal molecular and cellular endpoints before overt histopathological abnormalities are detectable. The findings support early changes in neuroprotective peptide signalling, UPR-associated gene expression, mitochondrial-dynamics-associated transcripts, neurotrophic regulation and astrocytic responses. These pathways were assessed in parallel, and the current data do not establish direct causal interactions among them. The results provide a foundation for longitudinal and mechanistic studies testing whether these early responses are reversible or progress to functional and structural injury.

## 4. Materials and Methods

### 4.1. Animals, Treatments and Tissue Isolation

All animal procedures were approved by the Animal Care and Ethics Committee of the University of Technology Sydney (UTS; ACEC #2018-3179) and conducted in accordance with the Australian National Health and Medical Research Council guidelines for the care and use of animals for scientific purposes. Male BALB/c mice aged 7 weeks were obtained from the Animal Resources Centre (Perth, WA, Australia) and maintained at 20 ± 2 °C under a 12:12 h light–dark cycle with ad libitum access to standard laboratory chow and water. Following acclimatisation, mice were randomly allocated to four experimental groups (n = 6 per group). Mice were exposed using an inExpose whole-body exposure system (SCIREQ Scientific Respiratory Equipment Inc., Montreal, QC, Canada) to the equivalent of 0, 2, 4 or 6 Winfield Red cigarettes per exposure session, with two sessions performed daily for four consecutive days. Exposure groups are designated throughout according to cigarette equivalents per session. Systemic exposure was confirmed by serum cotinine ELISA, yielding concentrations of 0.59 ± 0.21, 9.46 ± 2.39, 83.6 ± 10.7 and 295 ± 88 ng/mL in the 0-, 2-, 4- and 6-cigarette groups, respectively, as in previous work [63]. At the endpoint, mice were euthanised by the inhalation of 4% isoflurane delivered using 100% oxygen as the carrier gas (Veterinary Companies of Australia, Kings Park, NSW, Australia), with death confirmed by the absence of pedal and ocular reflexes before tissue collection. Brains were rapidly removed. One hemisphere was paraffin-embedded and sectioned coronally at 5 µm for histological and immunofluorescence analyses; the contralateral hippocampus was microdissected using the Paxinos & Franklin’s mouse brain atlas as a reference [64], and samples were stored at −80 °C for RT-qPCR or ELISA.

### 4.2. RNA Extraction, cDNA Synthesis and Real-Time Quantitative Polymerase Chain Reaction

Total RNA was extracted from hippocampal tissue using TRI Reagent according to the manufacturer’s instructions. RNA concentration and purity were assessed using an ND-1000 NanoDrop spectrophotometer. Samples were considered suitable when A260/A280 was greater than 1.8 and A260/A230 was greater than 2.0. RNA integrity was not independently assessed, and assay-specific amplification efficiencies were not determined. For each sample, 1 µg of total RNA was reverse transcribed using the Tetro cDNA Synthesis Kit. RT-qPCR was performed using a CFX96 Optics Module Real-Time System coupled to a C1000 Touch Thermal Cycler. The analysed genes were *Adcyap1*, *Vip*, *Adcyap1r1*, *Vipr1*, *Vipr2*, *Bdnf*, *Adnp*, *Mmp9*, *Sod1*, *Dnm1l*, *Opa1*, *Gfap*, *Ddit3*, *Ppp1r15a*, *Ern1*, *Eif2ak3*, *Il1b*, *Il6* and *Tnf*. *Rps18* was used as the reference gene for normalisation. Details are shown in Table 1. The retrospective analysis of animal-level *Rps18* Ct values detected no statistically significant differences among the four exposure groups within any of the four assay-associated datasets (one-way ANOVA, all *p* ≥ 0.132; n = 6 mice per group). Each 10 µL reaction contained 5 µL iTaq Universal SYBR Green Master Mix, 0.8 µL each of forward and reverse primers at 5 micromolar, 0.4 µL Milli-Q water and 3 µL cDNA. Reactions were performed in technical duplicate, and both no-template and no-reverse-transcriptase controls were included. Cycling comprised 95 °C for 2 min, followed by 45 cycles of 95 °C for 5 s, 60 °C for 10 s and 72 °C for 10 s, then followed by fluorescence acquisition and melting-curve analysis. A single melting peak was used to support gene-specific amplification. Relative expression was calculated using the 2^−ΔΔCt^ method [65] with *Rps18* as the reference and the control group as calibrator. Fold changes are displayed graphically, whereas statistical analyses were performed using ΔCt values.

### 4.3. Enzyme-Linked Immunosorbent Assay (ELISA)

Hippocampal concentrations of pituitary adenylate cyclase-activating polypeptide (PACAP) and vasoactive intestinal peptide (VIP) were quantified using commercially available mouse ELISA kits (MyBioSource, San Diego, CA, USA; PACAP: Cat. No. MBS2503456; VIP: Cat. No. MBS703048), as described in previous work [32].

Briefly, on the day of processing, snap-frozen hippocampi (n = 6 per group) were thawed, weighed and homogenised (1:10, *w*/*v*) in ice-cold phosphate-buffered saline (PBS) containing 0.5% Tween-20 supplemented with a freshly prepared protease and phosphatase inhibitor cocktail (1:100; Calbiochem, Merck Millipore, Sydney, NSW, Australia). Homogenates were centrifuged at 12,000× *g* for 25 min at 4 °C, and the clarified supernatants were collected for analysis. Total protein concentration was determined using the bicinchoninic acid (BCA) protein assay (Thermo Fisher Scientific, Waltham, MA, USA), according to the manufacturer’s instructions.

PACAP and VIP concentrations were quantified from the clarified lysates following the manufacturer’s protocols. Absorbance was measured at 450 nm using a Tecan Infinite^®^ M1000 PRO multimode microplate reader (Tecan Group Ltd., Männedorf, Switzerland). Peptide concentrations were calculated from standard curves generated using the supplied standards and normalised to total protein content. Data are presented as pg/mg protein.

### 4.4. Immunofluorescence

Immunofluorescence was performed on 5 µm paraffin-embedded coronal sections encompassing the dorsal hippocampus. Sections from control mice and mice exposed to the highest CS condition were analysed (0 and 6 cigarette equivalents per session, respectively; n = 6 mice per group). Sections were deparaffinised in xylene, rehydrated through graded ethanol solutions and subjected to heat-mediated antigen retrieval in 10 mM sodium citrate containing 0.05% Tween-20 at pH 6.0 for 20 min. Tissue autofluorescence was reduced using 0.25% ammonia in 70% ethanol for 1 h at room temperature. Sections were permeabilised in PBST containing 0.4% Triton X-100 for 30 min and blocked for 1 h in 5% bovine serum albumin containing 0.2% gelatine and 0.4% Triton X-100. Primary antibodies were applied overnight at 4 °C and included rabbit anti-phospho-IRE1α Ser724 (1:250; Cat. No. ZRB1072; Merck, Darmstadt, Germany), rabbit anti-BDNF (1:500; GeneTex, Irvine, CA, USA, Cat. No. GTX132621), rabbit anti-phospho-CREB Ser133 (1:250; Cell Signalling, Danvers, MA, USA, Cat. No. 9198), rabbit anti-GFAP (1:250, Abcam, Cambridge, UK, Cat No. ab68428) and mouse anti-betaIII-tubulin/TUJ1 (1:200, BioLegend, San Diego, CA, USA, Cat. No. 801201). Appropriate species-specific fluorescent secondary antibodies were applied for 1 h at room temperature in the dark. Sections were counterstained with DAPI and mounted using anti-fade fluorescence mounting medium.

### 4.5. Quantitative Image Analysis

Whole-slide fluorescence images were acquired using a Zeiss Axioscan Z1 digital slide scanner (Jena, Germany) with a 20× objective at a native spatial resolution of 0.33 micrometres per pixel. Images being directly compared were acquired using identical exposure and acquisition settings. Quantitative analysis was performed using Fiji/ImageJ ver. 1.52a by an investigator blinded to treatment allocation. Coded slides were used, and treatment identity was not revealed until quantification was complete. Corresponding control and exposed samples were analysed using consistent background-subtraction, thresholding and measurement parameters. Coronal sections encompassing comparable levels of the dorsal hippocampus were identified using consistent anatomical landmarks. For each mouse, three predefined FOVs were analysed: one in CA1, one in the contiguous CA2/3 field and one in DG. Each mouse therefore contributed one FOV-derived value per hippocampal subregion. FOV placement was based on anatomical and cytoarchitectural landmarks rather than staining intensity, and the mouse was the biological replicate (n = 6 per group).

For phospho-IRE1α immunolabelling, background signal was subtracted before application of a consistent intensity threshold. Immunoreactive cells were counted within each predefined FOV using Fiji/ImageJ and expressed as phospho-IRE1α-positive cells per FOV.

BDNF and phospho-CREB Ser133 immunoreactivity were quantified as background-corrected mean fluorescence intensity within the predefined CA1, CA2/3 and DG FOVs. Background fluorescence was determined from adjacent tissue lacking specific immunolabelling and subtracted from the corresponding measurement.

GFAP-positive cells were manually counted within each predefined FOV using Fiji/ImageJ. Cells were included when a clearly identifiable GFAP-positive soma with associated processes was visible. Results are expressed as GFAP-positive cells per FOV and are not stereological estimates of total astrocyte number or density within the entire hippocampal subregion.

### 4.6. Haematoxylin and Eosin Staining

Haematoxylin and eosin staining was performed on 5-micrometre coronal sections encompassing the dorsal hippocampus from the control and highest-exposure groups (n = 6 mice per group). Sections were deparaffinised in xylene, rehydrated through graded ethanol solutions, stained with Harris haematoxylin for 3 min, differentiated briefly in 1% acid alcohol and blued in Scott’s Tap Water Substitute Working Solution for 1 min. Sections were counterstained with 1% aqueous eosin for 2 min, dehydrated, cleared in xylene and mounted using DPX.

Histological assessment was qualitative and compared the overall hippocampal cytoarchitecture and neuronal morphology of sampled dorsal hippocampal sections between control and high-exposure animals. Because formal neuropathological scoring and unbiased stereology were not performed, the assessment was restricted to overt histological abnormalities and was not intended to exclude subtle cellular or structural alterations.

### 4.7. Statistical Analysis

Statistical analyses were performed using GraphPad Prism version 10.1.2 (GraphPad Software, Boston, MA, USA). Individual mice were considered as biological replicates, and data are presented as mean ± SEM with individual animal values shown where applicable. Technical RT-qPCR replicates were averaged before the calculation of animal-level values and were not treated as independent replicates. RT-qPCR expression is displayed as 2^−ΔΔCt^ fold change using Rps18 as the reference gene and the 0-cigarette group as calibrator; statistical analyses were performed on ΔCt values. Outcomes measured across all four exposure groups were analysed using ordinary one-way ANOVA with prespecified Dunnett comparisons of each exposed group with control. Normality of model residuals was assessed using the Shapiro–Wilk test and graphical inspection, and the homogeneity of variance was evaluated using the Brown–Forsythe test. Where unequal variances were detected, Brown–Forsythe and Welch ANOVA with variance-adjusted multiple comparisons was used. Linear trends were tested across the equally spaced exposure levels of 0, 2, 4 and 6 cigarettes per session. Two-group immunofluorescence comparisons used unpaired two-tailed *t*-tests. Selected transcript associations were examined using multiple linear regression with exposure level included as a covariate. The four prespecified predictor *p* values were corrected using the Benjamini–Hochberg false-discovery-rate procedure. These association analyses were considered exploratory and were not interpreted as evidence of causality. Statistical significance was defined as *p* < 0.05.

## Figures and Tables

**Table 1:** List of primers used in RT-qPCR analyses.

Accession Number	Gene Name	Primer Sequences	Size (bp)
NM_009625.3	Pituitary adenylate cyclase-activating polypeptide (PACAP; *Adcyap1*)	Fwd: GATCAGACCAGAAGACGAGG Rev: GCTGGATAGTAAAGGGCGTAAG	129
NM_011702.3	Vasoactive intestinal polypeptide (VIP; *Vip*)	Fwd: GACCACCTTCTGTAGTGAGTAG Rev: TTTCTGCTAAGGGATTCTGCAA	117
NM_007407.4	Pituitary adenylate cyclase-activating polypeptide 1 receptor 1 (PAC1; *Adcyap1r1*)	Fwd: TATCCACCATTACTCTACGGCT Rev: TCTGGAGAGAAGGCAAATACTG	91
NM_011703.4	Vasoactive intestinal peptide receptor 1 (VPAC1; *Vipr1*)	Fwd: CCCTCTGTTTGGAGTTCACTAT Rev: TACGACGAGTTCAAAGACCATT	88
NM_009511.2	Vasoactive intestinal peptide receptor 2 (VPAC2; *Vipr2*)	Fwd: ATTTCATAGATGCGTGTGGCTA Rev: TGCTTCCTGTTGTAAGAGACAT	126
NM_007540.4	Brain derived neurotrophic factor (BDNF; *Bdnf*)	Fwd: CGAGTGGGTCACAGCGGCAG Rev: GCCCCTGCAGCCTTCCTTGG	160
NM_001310086.1	Activity-dependent neuroprotective protein (ADNP; *Adnp*)	Fwd: GTGACATTGGGTTGGAATACTGT Rev: AGGTTTTGTCCGATAGTCCTGA	149
NM_013599.4	Matrix metallopeptidase 9 (MMP9; *Mmp9*)	Fwd: ATCATAGAGGAAGCCCATTACAG Rev: TTTGACGTCCAGAGAAGAAGAAA	129
NM_011434.2	Superoxide Dismutase 1 (SOD1; *Sod1*)	Fwd: CAATGGTGGTCCATGAGAAACA Rev: CCCAGCATTTCCAGTCTTTGTA	77
NM_001360007.1	Dynamin-Related Protein 1 (DRP1; *Dnm1l*)	Fwd: GCAGAGGATCATTCAGCATTGTA Rev: TTGCCACTAAGTTATGCACCATT	149
NM_001199177.1	Optic Atrophy 1 (OPA1; *Opa1*)	Fwd: GCCCTTCTCTTGTTAGGTTCAC Rev: ACACCTTCCTGTAATGCTTGTC	88
NM_001131020.1	Glial Fibrillary Acidic Protein (GFAP; *Gfap*)	Fwd: GAGATTCGCACTCAATACGAGG Rev: CTGCAAACTTAGACCGATACCA	79
NM_007837.4	C/EBP Homologous Protein (CHOP; *Ddit3*)	Fwd: GCTCTCCAGATTCCAGTCAG Rev: CTCCTTCTCCTTCATGCGTT	131
NM_008654.2	Growth Arrest and DNA Damage-Inducible Protein 34 (GADD34; *Ppp1r15a*)	Fwd: AGAAGGAAATGGAACGCACA Rev: CCAGCTGTGATGTGGGATAA	135
NM_023913.2	Inositol-requiring enzyme 1 (IRE1; *Ern1*)	Fwd: GAGACAAAGGAGAGTGTGTGAT Rev: TCAAGTAGTTCAGCTTGCTCTT	87
NM_010121.3	Protein kinase RNA-like endoplasmic reticulum kinase (PERK; *Eif2ak3*)	Fwd: CCTTGGTTTCATCTAGCCTCA Rev: ACTTGTAGGAAGATTCGAGCAG	156
NM_008361.4	Interleukin 1 beta (IL-1β; *Il1b*)	Fwd: GCTACCTGTGTCTTTCCCGT Rev: CATCTCGGAGCCTGTAGTGC	164
NM_031168.2	Interleukin 6 (IL-6; *Il6*)	Fwd: CCCCAATTTCCAATGCTCTCC Rev: CGCACTAGGTTTGCCGAGTA	141
NM_013693.3	Tumour necrosis factor (TNF; *Tnf*)	Fwd: ATGGCCTCCCTCTCATCAGT Rev: TTTGCTACGACGTGGGCTAC	97
NM_011296.2	Ribosomal protein S18 (RPS18; *Rps18*)	Fwd: CCCTGAGAAGTTCCAGCACA Rev: GGTGAGGTCGATGTCTGCTT	145

## Data Availability

The original contributions presented in this study are included in the article/supplementary material. Further inquiries can be directed to the corresponding author.

## Supplementary Materials

The following supporting information can be downloaded at: https://www.mdpi.com/article/10.3390/ijms27198760/s1.
