# Supplementary material for: Early Hippocampal Stress Responses to Short-Term Cigarette Smoke Exposure in Mice
Source: Int J Mol Sci. 2026 Sep 30;27(19):8760. doi: 10.3390/ijms27198760 (PMC13664968; doi:10.3390/ijms27198760)
Supplement: Supplementary file 1 [file ijms-27-08760-s001.zip › Supplementary Figure S1.docx]

**Supplementary File S1**

**Supplementary Figure S1. Short-term cigarette smoke exposure does not produce significant group-wise changes in hippocampal pro-inflammatory cytokine gene expression. Relative hippocampal expression of *Il1b* (A), *Il6* (B) and *Tnf* (C) was measured by RT-qPCR after exposure to 0, 2, 4 or 6 cigarettes per session, twice daily for four consecutive days. Fold-change values calculated using the 2^-ΔΔCt^ method are displayed as mean ± SEM with individual mice shown (n=6 per group), whereas statistical analyses were performed on ΔCt values. Group differences were assessed using one-way ANOVA followed by Dunnett comparisons with control, and linear trends were tested across the ordered exposure levels. No individual exposure group differed significantly from control for any transcript. *Il1b* showed a significant exploratory linear trend (Ptrend = 0.023), whereas *Il6* and *Tnf* showed no significant linear trends. ns, not significant.**
