# Supplementary material for: Early Hippocampal Stress Responses to Short-Term Cigarette Smoke Exposure in Mice
Source: Int J Mol Sci. 2026 Sep 30;27(19):8760. doi: 10.3390/ijms27198760 (PMC13664968; doi:10.3390/ijms27198760)
Supplement: Supplementary file 1 [file ijms-27-08760-s001.zip › Supplementary Table S1.docx]

**Supplementary Table S1**

**Exposure-adjusted regression results for the four prespecified transcript associations**

| **Predictor** | **Outcome** | **β** | **SE** | **95% CI** | **t** | **p** | **BH q** | **Adjusted R²** |
| --- | --- | --- | --- | --- | --- | --- | --- | --- |
| *Dnm1l* | *Mmp9* | −0.043 | 0.199 | −0.456 to 0.370 | −0.215 | 0.832 | 0.832 | 0.613 |
| *Opa1* | *Mmp9* | −0.037 | 0.140 | −0.328 to 0.253 | −0.267 | 0.792 | 0.832 | 0.613 |
| *Dnm1l* | *Opa1* | 0.512 | 0.289 | −0.090 to 1.113 | 1.769 | 0.091 | 0.183 | 0.727 |
| *Sod1* | *Mmp9* | 0.290 | 0.118 | 0.045 to 0.535 | 2.466 | 0.022 | 0.090 | 0.699 |

**Notes.** Each multiple linear regression model included exposure level (0, 2, 4 or 6 cigarettes per session) as a covariate. Analyses were performed on ΔCt values (n = 24; residual df = 21). β values are unstandardised coefficients for the indicated transcript predictor. Because a higher ΔCt represents lower relative expression, coefficient direction should be interpreted on the ΔCt scale. The p value tests the predictor coefficient; q values were calculated using the Benjamini–Hochberg false-discovery-rate procedure across the four prespecified predictor tests. None of the transcript associations remained significant after FDR correction (q < 0.05). CI, confidence interval; SE, standard error.
